# Enterohemorrhagic *Escherichia coli* and a Fresh View on Shiga Toxin-Binding Glycosphingolipids of Primary Human Kidney and Colon Epithelial Cells and Their Toxin Susceptibility

**DOI:** 10.3390/ijms23136884

**Published:** 2022-06-21

**Authors:** Johanna Detzner, Gottfried Pohlentz, Johannes Müthing

**Affiliations:** Institute of Hygiene, University of Münster, D-48149 Münster, Germany; johanna.detzner@ukmuenster.de

**Keywords:** detergent-resistant membranes, EHEC, glycolipids, lipid rafts, STEC, Stx1a, Stx2a

## Abstract

Enterohemorrhagic *Escherichia coli* (EHEC) are the human pathogenic subset of Shiga toxin (Stx)-producing *E. coli* (STEC). EHEC are responsible for severe colon infections associated with life-threatening extraintestinal complications such as the hemolytic-uremic syndrome (HUS) and neurological disturbances. Endothelial cells in various human organs are renowned targets of Stx, whereas the role of epithelial cells of colon and kidneys in the infection process has been and is still a matter of debate. This review shortly addresses the clinical impact of EHEC infections, novel aspects of vesicular package of Stx in the intestine and the blood stream as well as Stx-mediated extraintestinal complications and therapeutic options. Here follows a compilation of the Stx-binding glycosphingolipids (GSLs), globotriaosylceramide (Gb3Cer) and globotetraosylceramide (Gb4Cer) and their various lipoforms present in primary human kidney and colon epithelial cells and their distribution in lipid raft-analog membrane preparations. The last issues are the high and extremely low susceptibility of primary renal and colonic epithelial cells, respectively, suggesting a large resilience of the intestinal epithelium against the human-pathogenic Stx1a- and Stx2a-subtypes due to the low content of the high-affinity Stx-receptor Gb3Cer in colon epithelial cells. The review closes with a brief outlook on future challenges of Stx research.

## 1. Introduction

A wide range of bacterial AB_5_-toxins is known for a long time having the ability to enter mammalian cells via protein-carbohydrate interactions videlicet binding of soluble toxins of the pathogen to glycans exposed on the mammalian cell surface [1,2]. Numerous glycan-recognizing harmful proteinous virulence factors (by definition lectins) produced by pathogenic bacteria have been scrutinized in the past decades upon their discovery, and their molecular mechanisms by which they cause diseases have been largely unraveled since that time [3,4,5,6]. Examples of renowned sugar-binding AB_5_ toxin specimens are the long known cholera toxin of *Vibrio cholerae* [7,8,9], the heat-labile enterotoxins of *Escherichia coli* [10,11,12], subtilase cytotoxin from *E. coli* [4,13,14,15,16,17,18], and Shiga toxins (Stxs) from *Shigella dysenteriae* and *E. coli* [19,20,21,22].

Human endothelial cells of various endothelial beds are well known targets of Stxs, while the role of kidney and colon epithelial cells in EHEC-caused disease is still a matter of debate. In this review we will report on the state of research regarding the interaction of the two human-pathogenic Stx-subtypes Stx1a and Stx2a with human kidney and colon epithelial cells putting the focus on primary human renal proximal tubular epithelial cells (pHRPTEpiCs) and primary human colon epithelial cells (pHCoEpiCs). The lower case letter “p” stands for “primary” and emphasizes the fact that normal healthy cells are covered in this review, whereas tumor-derived epithelial cells and virus-transformed or otherwise immortalized epithelial cells are not considered here.

The first part what we report about is the clinical impact of enterohemorrhagic *E. coli* (EHEC), the human-pathogenic subgroup of Stx-producing *E. coli* (STEC). The subsequent explanations of the Stx-mediated cytotoxic activity refer particularly to kidney and colon epithelial cells and include novel findings regarding the involvement of microvesicles in Stx-associated infection and the vesicular transport of Stx in the human bloodstream backed up with the latest publications. Next we provide some general remarks on the structures of Stx and glycosphingolipids, followed by a section in which the detection of Stx-binding glycosphingolipids and their detailed lipoforms determined in pHRPTEpiCs and pHCoEpiCs is dealt with based on very recent own investigations. This part of the review is supplemented with few short comments to the highly efficient procedure for affinity-purification of Stxs and their mass spectrometric identification by diagnostic ions. We then set out the occurrence of Stx receptor glycosphingolipids in membrane microdomains (known as lipid rafts) of pHRPTEpiCs and pHCoEpiCs, respectively, using detergent-resistant membranes as analogs of the liquid-ordered membrane phase including also some general remarks on the use of this biochemical methodology. The descriptions continue with comparative data on the different susceptibility of pHRPTEpiCs and pHCoEpiCs toward the human-pathogenic Stx1a and Stx2a subtypes. This section is followed by a presentation of the current status regarding therapeutic options of EHEC infections. The review closes with an outlook on groundbreaking improvements obtained by imaging mass spectrometry showing the potential of the in situ visualization of the various lipoforms of all kinds of lipids in tissue sections, and the increasing number of newly developed glyco-derivatives and promising alternative strategies aimed at neutralization or at least mitigation of the cytotoxic action of Stxs.

## 2. Clinical Impact of Colonic EHEC Infections, Stx-Mediated Extraintestinal Complications, and Organ Damage

This section provides a survey of the clinical impact of EHEC infections including the pathogen’s epidemiology and virulence potency. The topics described first are the colonization of EHEC bacteria in the gut and new insights about the release of EHEC-derived virulence factors entrapped in or associated with outer membrane vesicles with focus on Stx. New findings about the possible mode(s) of translocation of Stx from the gut into the blood and the toxin’s transportation in the circulation delineate the penultimate aspects of this section, which closes with brief remarks of EHEC-caused extraintestinal complications.

### 2.1. EHEC Zoonotic Infections and Reservoir

Humans usually become infected through the ingestion of food (mostly ground beef, leaf vegetables, and sprouts) or water contaminated with EHEC derived from ruminant feces [23,24,25,26]. In the past few years, however, a number of new animal species from wildlife and aquaculture industries have also been identified as unexpected origins of zoonotic STEC infections [27]. Among ruminants, cattle are the environmental priority reservoir of EHEC with shedding varying greatly among individuals and highly variable, but unpredictable pathogenic potential for humans [28,29,30,31,32]. Importantly, an animal reservoir of the Stx-producing 2011 German *E. coli* O104:H4 outbreak strain is presently unknown. Pigs and poultry are not considered to be the source of EHEC although conflicting evidence on the role that swine play in the transmission of STEC to people and human illness requires further evaluation [33,34]. Interestingly, the Stx2e subtype is responsible for porcine edema disease, which is the sole disease caused by STEC in animals [35,36,37]. EHEC can colonize the animals for several months serving as gene reservoirs for the genesis of highly virulent zoonotic EHEC strains questioning our current understanding of the molecular basis of adaptation of this important *E. coli* pathovar [38,39]. Whether cattle represent asymptomatic carriers as persistent colonizers of the gut without any signs of infections or not is a matter of debate [25]. Upon colonization of the ruminant large intestine, EHEC may especially target follicle-associated epithelial cells in the terminal rectum and induce attaching and effacing (A/E) lesions, mediated by proteins released by the type III secretion system (T3SS) and the outer membrane protein intimin [40]. Evidence has been provided that many EHEC strains, which cause A/E lesions or at least carry genes for this trait, are diarrheagenic pathogens of calves [41]. Furthermore, cattle-derived peripheral and intestinal lymphocytes, certain other colonic epithelial cells and macrophage-like cells have been identified in the past as possible targets for EHEC [25,42,43,44,45]. Furthermore, the Stx receptor glycosphingolipids of the globo-series (more precisely dealt with in Section 4) have been detected in the bovine small and large intestine as well as discrete cell subsets in the bovine kidney and submucosal lymphoid cells, but not in the vasculature [46]. Thus, the proven absence of Stx receptors on bovine vascular endothelium is possibly the reason for the apparent resistance of cattle to systemic effects of Stx and provides the explanation for the low damage potential of Stx toward bovine aortic endothelial cells [47] known to harbor only traces of Stx glycosphingolipid receptors [48]. From this it can be concluded that the diverse cellular repertoire of Stx receptors might be the reason for the distinct Stx-mediated effects in cattle versus those implicated in EHEC-caused human diseases [25].

### 2.2. Epidemiology and Virulence Potency of EHEC

**Figure 1:**
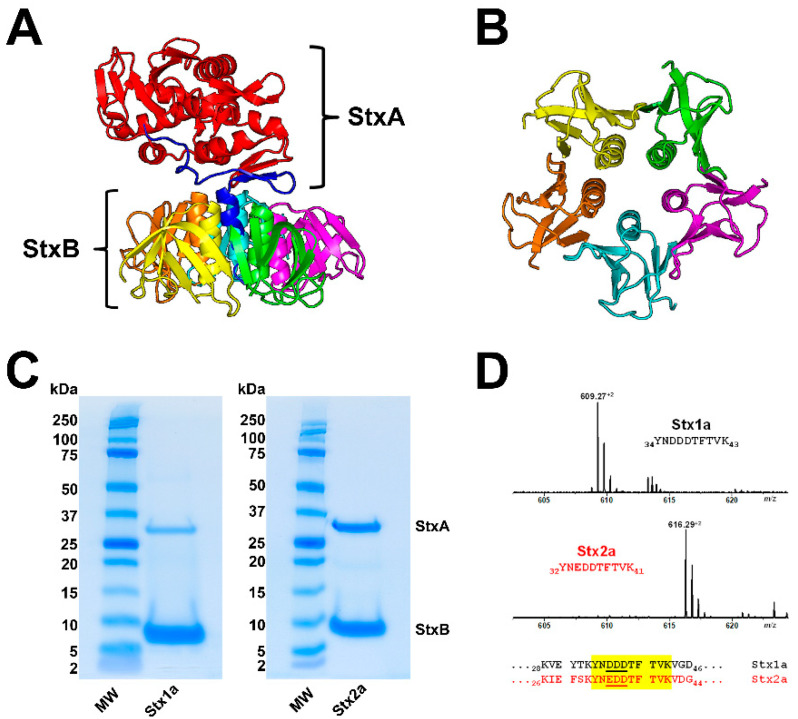
Ribbon diagrams of the Stx2a crystal structure (**A**) and the homopentameric StxB viewed from below (**B**); SDS-PAGE-separated affinity-purified Stx1a and Stx2a (**C**) and signals of Stx1a and Stx2a subtype-specific diagnostic peptide ions detected by mass spectrometry (**D**). The structure of Stx2a (1R4Q) was drawn with PyMOL Molecular Graphics System, Version 2.0 (Schrödinger, Synaptic Science, LLC, Bethesda, MD, USA) based on the amino acid sequence published by Fraser and co-workers [171] deposited in the Research Collaboratory for Structural Bioinformatics (RCBS) data bank (https://www.rcsb.org/structure/1R4Q, 5 June 2022). The StxA subunits (StxA) and StxB subunits (StxB) were separated under reducing conditions and stained with Coomassie Blue (**C**). Diagnostic peptide ions derived from tryptic digestions of the B subunits of Stx1a and Stx2a, the peptide positions and their amino acid sequences within the B subunits are highlighted in yellow (taken from Steil and co-workers [170].

An estimated 470 STEC serotypes have been identified, which can produce one or more of the 12 known Stx subtypes [49]. EHEC isolates produce mostly Stx1a and/or Stx2a subtypes (for Stx structure see Figure 1) corresponding to the toxins in the literature often incorrectly designated as Stx1- and/or Stx2-subtypes or named as vero(cyto)toxin 1 (VT1) and VT2, or Shiga-like toxin (SLT1) and SLT2, respectively (for accurate designation of the various Stx-subtypes refer to Scheutz and collaborators) [50]. The well-known Stxs released by *E. coli* O157:H7 and other serotypes are currently the best characterized virulence factors of EHEC strains. *E. coli* O157:H7 is the most frequently identified EHEC serotype in patients with HUS worldwide and the number of STEC serotypes that cause human illness is probably higher than 100 [25,49,51,52,53]. However, in 2011 Germany experienced the historically largest clonal outbreak with an Stx-producing *E. coli* strain of O104:H4 serotype ever recorded spreading to Northern Europe and illustrating the emerging importance of this non-O157 EHEC strain documented with 855 HUS cases and 53 deaths [54,55,56,57,58,59,60,61]. Although serotype information is useful in outbreak investigation and surveillance studies, it is not a reliable means of assessing the human health risk by a particulate STEC serotype [49].

The clinical significance with regard to the risk of developing severe diseases as a consequence of an EHEC infection varies considerably with the different Stx1- and Stx2-subtypes [20,62,63,64], which are associated with the EHEC-caused hemolytic-uremic syndrome (HUS) to varying degrees [65,66,67,68]. Among the various subtypes, Stx2a is considered to be the epidemiologically more important one than Stx1a with respect to the development of HUS [69]. *E. coli* O157:H7 strains harboring the *stx2a* gene exhibit higher virulence potency and are more frequently associated with HUS [70]. In general, it was shown that patients infected with EHEC strains carrying *stx2a* as the sole *stx* gene, have been found to develop HUS significantly more frequently than those infected with strains harboring *stx1a* only or *stx1a* together with *stx2a* [65,71]. The reason for differential toxicity of Stx1(a) and Stx2(a) could be the stronger ribosomal affinity and higher catalytic activity of the A1 fragment of the A subunit of Stx2(a) compared to Stx1(a) (for Stx structure see Figure 1 in Section 3) [72]. Last but not least, it should be noted that the *stx2a* gene is most often present in STEC strains positive for the locus of enterocyte effacement (LEE) and has consistently been associated with HUS [49]. It is assumed that the Stx genotype and perhaps known and/or yet unknown additional virulence factors rather than the amount of Stx or the in vitro cytotoxicity may correlate with the appearance of HUS [73]. However, the importance of the Stx amount in the development of HUS seems to be underestimated, because a combination of both criteria, namely the Stx genotype and the Stx production level, is more likely to contribute to EHEC pathogenicity. Studies showed that hypervirulent lineage of EHEC O157:H7 (clade 8) carried an Stx2a phage subtype conferring the highest Stx2a production to the host strain [74] and the types of Stx phage replication proteins, which influence Stx production, correlated with EHEC virulence potential [75]. Furthermore, one must be aware that some serotypes of EHEC can produce additional virulence factors besides Stx including cytolethal distending toxin [76,77,78,79,80,81], EHEC hemolysin [82,83,84] and subtilase cytotoxin [4,15,16,17,18,85,86,87,88], which might have a cumulative effect as single compounds or as “cocktails” on Stx-mediated cellular injury.

### 2.3. EHEC Colonization of the Gut

EHEC can colonize the human intestinal tract being responsible for severe intestinal illnesses [89,90,91]. Moreover, EHEC affect the interaction between the host and the commensal microbial communities and influence the diversity of the indigenous gut microbiota [92,93,94,95,96,97]. In the gut, EHEC induce A/E lesions at the apical surface of enterocytes of the host colon [98,99]. The induction of A/E lesions is mediated by virulence factors of the T3SS located in a genome-inserted large pathogenicity island LEE [100,101,102,103]. LEE genes encode for the adhesive protein intimin, its bacterially encoded receptor Tir, and effector proteins, which are secreted through a T3SS from the bacterial cytosol into the infected cells [104,105,106]. Regrouping of the actin cytoskeleton underneath the attached bacteria accompanies the formation of A/E lesions and results in the formation of pedestals leading to destruction of the microvilli of the brush border [101,107,108,109]. Importantly, an animal infection model that fully recapitulates EHEC-caused human diseases remains elusive [110].

### 2.4. Colonic Outer-Membrane Vesicles of EHEC

In the vast majority of *E. coli* strains the genes encoding for Stxs are located in the genome of heterogenous lambdoid prophages and are under control of phage genes [111,112,113]. Upon induction, the prophage can switch from the lysogenic state to the lytic cycle, accompanied by production of large amounts of new phage particles, which is tightly linked to synthesis and subsequent release of Stx when the host cell bursts [114,115]. Spontaneous chemical or physical induction results in co-transcription of *stx*-genes together with the late phase genes of the prophages, and after cell lysis mature bacteriophage particles together with Stx are released into the environment [116]. Stx-releasing EHEC O157:H7 and O104:H4 strains have been shown to shed nanoscale outer membrane vesicles (OMVs) into the culture medium. Such OMVs contain DNA encoding for a number of virulence genes and are loaded with EHEC-descendent virulence factors including Stx1(a) and Stx2(a) [117,118]. The OMVs of the O104:H4 outbreak strain and EHEC O157 clinical isolates were found to contain a cocktail of virulence determinants besides the key OMV component Stx2a and to bind to human intestinal epithelial cells, followed by internalization and delivery of the OMV-associated virulence factors to the interior of the cells following different intracellular retrograde routes [118,119,120]. Such OMVs are important tools for pathogenic *E. coli* to deliver pathogenic cargoes and injure host cells, whereby antibiotic-mediated and intrahost milieu-dependent increase of OMV production has been reported for EHEC O157:H7 and O104:H4 that might worsen the clinical outcome of infections [121,122]. Originally dismissed as an artifact of the cell wall due to release of cell debris by decaying bacteria, OMVs are now considered as a general bacterial secretion system and “long distance weapons” that contribute significantly to the virulence of pathogenic bacteria [123].

### 2.5. Translocation of Shiga Toxin and Toxin Carriers in the Circulation

To reach the major target cells especially sensitive endothelial cells lining the microvasculature of kidneys and brain, Stx must cross the intestinal epithelial barrier to enter the blood stream and systemically spread into the circulation. In vitro models have been developed to study the passage of Stx through polarized monolayers using the human colonic epithelial cell lines CaCo-2, T84, and HCT-8 [124,125,126,127,128,129,130]. Biologically active Stx was found being capable of moving across the epithelial layer of T84 cells most likely via a transcellular route without apparent cellular disruption as assessed by measuring transmonolayer electrical resistance [131]. Using a vertical diffusion chamber system with T84 cells and simulating an intestinal microaerobic environment [132], microaerobiosis caused significant reduction of Stx production and decreased release into the medium, whereas Stx translocation across the T84 epithelial monolayer was enhanced under microaerobic environment versus aerobic conditions [133]. Interestingly, the EHEC O157:H7 and O104:H4 serotypes showed considerable differences in colonization, Stx production, and Stx translocation suggesting alternative virulence strategies [133].

**Figure 2:**
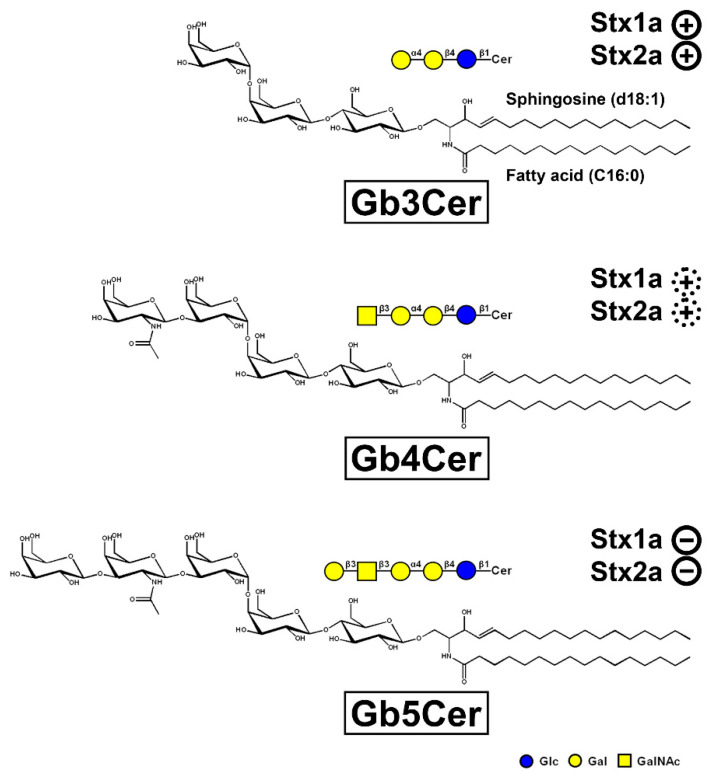
Tri-, tetra-, and pentahexosylceramides of the globo-series. The three GSLs are exemplarily portrayed with a ceramide (Cer) moiety carrying sphingosine (d18:1) and a C16:0 fatty acyl chain. The GSLs were drawn with the program ChemDraw 2019, Version 19.1.1.21 (PerkinElmer Informatices, Inc., Waltham, MA, USA) and the sugars are depicted in the chair conformation. Declining adhesion strength of Stx1a and Stx2a along with increasing sugar chain length of the globo-series glycans is indicated: strong binding of Stx1a and Stx2a to Gb3Cer (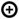), weak binding to Gb4Cer (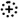), and no binding at all to Gb5Cer (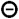).

In the blood stream, binding of Stx toward polymorphonuclear leukocytes may occur, a process that has been shown by in vitro studies [134,135] and underlined by detection of the transfer of cell-bound Stx to glomerular microvascular endothelial cells [134]. The possible functional role of neutrophils as toxin carriers for the transfer of Stx to kidney endothelium and consequently the development of HUS was further supported by proof of Stx-loaded polymorphonuclear leukocytes in the systemic circulation of patients with HUS [136] and of persons among households with children with HUS [137]. Moreover, the immunodetection of Stxs on circulating neutrophils in the blood of children with HUS has been identified as a valuable tool for laboratory diagnosis and confirms once again the role of neutrophils in the pathogenesis of this syndrome [138]. Experiments using confluent endothelial cell monolayers and Stx-loaded polymorphonuclear leukocytes in a two-chamber transmigration device that mimics the toxin-induced endothelial injury further corroborated the role of neutrophils as toxin carriers [139]. Interestingly, conformational changes of Stx due to reduced α-helix content, which may occur throughout complicated and long-lasting multi-step purification procedures, resulted in loss of neutrophil binding of the toxin [140]. Toll-like receptor 4 (TLR4) is the receptor of Stx on human neutrophils, which is specifically recognized by Stx1(a) and Stx2(a) independently of the canonical Stx-binding GSL globotriaosylceramide (Gb3Cer) [141]. Gb3Cer (for structure see Figure 2), the renowned recognition motif of the B pentamer of Stxs, is not expressed by neutrophils and the interplay between TLR4 and Stx is based on a protein–protein interaction likely between the Stx A subunit and TLR4. TLR4 facilitates binding of Stx to colon carcinoma and primary human umbilical vein endothelial cells [142]. Soluble TLR4 has been reported to infringe the capture of Stx2a by human serum amyloid P component—a negative modulating factor that specifically binds Stx2a and impairs its toxic action—allowing the toxin to target and damage human cells suggesting TLR4 as a positive modulating factor for Stx2a [143]. Among non-cellular blood constituents extracellular microvesicles shed from platelets and blood cells may participate in the transfer of Stx from the circulation into the kidney and are suggested to be involved in Stx-associated HUS, thrombosis, hemolysis and renal failure [144]. Stx can reach the kidney within microvesicles and only recipient cells possessing endogenous Gb3Cer were found to exhibit cellular injury after uptake of Stx-bearing Gb3Cer-positive microvesicles [145]. In addition, increasing evidence has been provided that extracellular Stx2(a)-bearing vesicles released in the blood of patients by toxin-challenged circulating cells (monocytes, neutrophils, and erythrocytes) and platelets are key factors in targeting renal endothelial cells and thus in the pathogenesis of HUS [146,147].

### 2.6. EHEC-Caused Systemic Complications

EHEC infections of humans start with watery and bloody diarrhea upon colonization, may be involved in bowel necrosis, colonic perforation, and intussusceptions and can manifest as serious hemorrhagic colitis [92,148,149,150,151,152]. The hallmark of hemorrhagic colitis due to EHEC is the development of bloody diarrhea several days after the onset of non-bloody diarrhea and abdominal pain. More severe EHEC infections can progress to systemic life-threatening complications, such as HUS and neurological abnormalities of the central nervous system [21,62,63,152,153,154,155]. Within a week of onset of EHEC-mediated diarrhea/colitis, HUS develops abruptly and manifests as thrombocytopenia, intravascular microangiopathic hemolytic anemia, and acute renal insufficiency with kidney as the most commonly affected organ [152,156,157,158,159,160]. However, serious cerebral dysfunctions such as altered mental status, seizures, stroke, and coma are most common cause of acute mortality in HUS patients of EHEC infections [152,161]. About 70% of patients have been reported in long-term outcomes of EHEC-HUS that recovered fully from the acute phase, while the remainder showed varying degrees of post-diarrheal sequelae including proteinuria, hypertension, decreased glomerular filtration rate, or neurological symptoms [152,162,163].

## 3. Shiga Toxin Structure and Glycosphingolipid Receptor Lipoforms

### 3.1. Shiga Toxin Structure

Stx is a classic representative of the AB_5_ toxins composed of a single A subunit (StxA, 32 kDa), where “A” stands for “activity”, and five identical B subunits (StxB, 7.7 kDa each), where “B” stands for “binding” [4,20,164] as shown in Figure 1A,B. The catalytic A subunit of the ribosome-inactivating protein exerts interruption of the protein biosynthesis at the ribosomes and the pentameric B subunit binds to globo-series GSLs on the target cell surface [70,165,166,167]. Stxs are grouped in the two types Stx1 and Stx2, which are further subdivided into three Stx1 and (at least until now) nine Stx2 subtypes (for correct nomenclature refer to Scheutz and collaborators) [50]. Stx produced by *Shigella dysenteriae* type 1 is almost identical to Stx1 of STEC (differing in only one amino acid in the A subunit), and Stx1 and Stx2 share 55% amino acid homology [168], more precisely 55% for the A subunit and 57% for the B subunits [20]. The Stx1 subtypes are Stx1a, Stx1c, and Stx1d, and those of Stx2 are Stx2a, Stx2c to Stx2i and the recently discovered Stx2k [169]. The Cooomoassie Blue-stain of SDS-PAGE-separated Stx1a and Stx2a (Figure 1C) shows their 32 kDa-sized A subunits (StxA) and 7.7 kDa-sized B subunits (StxB). Highly purified Stx1a and Stx2a were obtained by affinity-purification, a highly efficient and practicable procedure working on small volume scale using Gb3-functionalized magnetic beads which makes this procedure superior to any time-consuming and labor-intensive multistep column chromatographies [170]. Subtype verification can be done by mass spectrometry (MS) detecting and sequencing the Stx1a- and Stx2a-specific diagnostic peptide ions in the *m*/*z* range between 608 and 618 as shown in Figure 1D. Doubly charged ions derived from the tryptic decapeptides _34_YNDDDTFTVK_43_ of Stx1a at *m*/*z* 609.27 and _32_YNEDDTFTVK_41_ of Stx2a at *m*/*z* 616.29 represent diagnostic ions for the respective Stx subtype and are suitable for a fast and facile MS-based identification of Stx subtypes.

### 3.2. Glycosphingolipid Receptor Lipoforms

GSLs are amphipathic molecules that are composed of a hydrophilic glycan moiety and a hydrophobic twin-tailed ceramide (Cer), which is built up from a long-chain amino alcohol and a fatty acyl chain [172,173,174,175,176,177,178]. While sphingosine (d18:1; sphingenine) represents the most common base as a constant part of GSLs’ ceramide, the fatty acyl chain can vary in chain length from C14 to C26 leading to various GSL lipoforms. It was the German physician Johann Ludwig Wilhelm Thudichum, who coined the term “sphingosine” in 1884 for its enigmatic “Sphinx-like” properties [172,179]. In Greek mythology, the sphinx is a monster that posed a riddle to all it encountered and destroyed those who could not answer the riddle [179]. Today, (glyco)sphingolipids are the subjects of intense studies aimed at elucidating their role in the cell membrane, their participation in signaling and recognition events, and especially their involvement in pathological processes being the basis for numerous human diseases [172,174,175,176,177,178].

Hemorrhagic colitis and life-threatening HUS caused by EHEC is triggered by Stx, the HUS-associated key virulence factor of this pathogen. The Stx subtypes Stx1a and/or Stx2a released by EHEC are of central importance in the development of the disease recognizing globo-series GSLs on the long known target cells videlicet endothelial cells of kidney and brain [165,166,180]. Stx1a and Stx2a bind with high priority to globotriaosylceramide (Gb3Cer, Galα4Galβ4Glcβ1Cer), to much lesser extent to globotetraosylceramide (Gb4Cer, GalNAcβ3Galα4Galβ4Glcβ1Cer), and not at all to Gb5Cer (Galβ3GalNAcβ3Galα4Galβ4Glcβ1Cer) as exemplarily shown in Figure 2 for the lipoforms harboring sphingosine (d18:1) and a short-chain fatty acid (C16:0) in the ceramide portion. It is obvious that the stepwise elongation of the oligosaccharide moiety of Gb3Cer to Gb4Cer and to Gb5Cer goes along with rapidly decreasing attachment of Stx1a and Stx2a (Figure 2). The prevalent lipoforms of Gb3Cer and Gb4Cer determined in the past for primary human endothelial cells of brain and kidney as well as erythroblast target cells are those with Cer (d18:1, C16:0), Cer (d18:1, C22:0), and Cer (d18:1, C24:1/C24:0) [166,181,182,183,184]. One of the most obvious “mysteries” is this enigmatic fatty acyl chain heterogeneity with unknown biological relevance regarding this variability in chain length, in particular the species with C24:0/C24:1 fatty acyl chains. The involvement of C24:1/C24:0 fatty acid-containing lactosylceramide (Lc2Cer) in Lc2Cer-mediated superoxide generation and migration in neutrophils suggested a functional role of this Lc2Cer lipoform in basic neutrophilic signaling processes [185]. In addition, interaction between C24 fatty acid-bearing Lc2Cer of neutrophil-like cells (neutrophilic differentiated HL-60 cells) and the Src family kinase Lyn has been shown as an instrumental requirement for signaling processes [186]. Furthermore, an interdigitation between the C24 fatty acyl chain of sphingomyelin in the outer leaflet of the plasma membrane with the glycerophospholipid phosphatidylserine (18:0/18:1) in the inner leaflet of the plasma membrane has been described, a process which is known as “handshaking” and hypothesized as important mechanism in membrane biology [187,188].

## 4. Shiga Toxin Receptor Glycosphingolipids of Primary Human Kidney and Colon Epithelial Cells

The cascade leading from gastrointestinal infection to renal impairment is complex, with the microvascular endothelium of kidneys and brain being the major histopathological target [62,166,189,190,191]. In addition, the observed Stx-mediated toxicity toward developing erythrocytes (erythroblasts) determined in an ex vivo human erythropoiesis model [181,184] and the susceptibility of kidney and colon epithelial cells toward Stx make the situation even more complicated. Data on the GSL profiles with respect to Stx-binding globo-series GSLs of human kidney and colon epithelial cells have been reported in the past by various expert research groups. Their preliminary results regarding the structures of the Stx receptor GSLs published in earlier times were appreciated by citations in our previous articles, where we addressed this issue and published for the first time the full structures of Stx receptors of primary human kidney epithelial cells [192,193] and colon epithelial cells [194]. The GSL profiles of kidney- and colon-derived tumor cell lines or otherwise immortalized cells are largely influenced by the tumor type itself and/or the developmental stage of the tumor, where the cell lines descend from, as well as the immortalization vector (discussed in more detail in the context of cell susceptibility toward Stx in Section 6 below). These cell lines have been described to express different or even complete opposite GSL repertoires compared to primary cells, e.g., high Gb3Cer levels versus Gb3Cer negative GSL profiles, which indicate great discrepancies toward primary cells. This excludes immortal cell lines from reliable investigations in comparison to primary cells derived from healthy human organs reflecting more authentically the human in vivo situation. The advantages of primary cells have been explained in greater detail in a previous review of Legros and co-workers [166].

**Figure 3:**
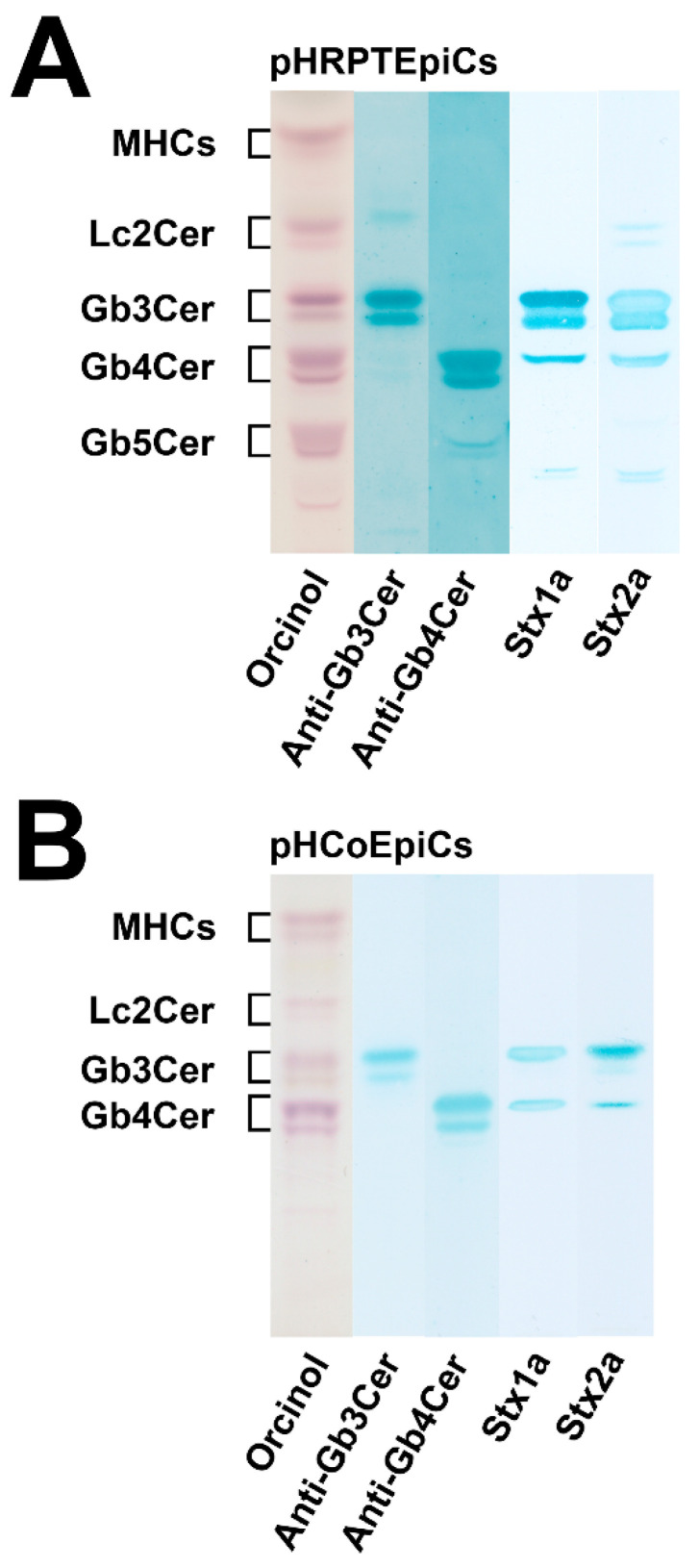
Orcinol stain and anti-Gb3Cer, anti-Gb4Cer, Stx1a and Stx2a TLC overlay assays of the neutral GSL preparations of pHRPTEpiCs (**A**) and pHCoEpiCs (**B**). Applied GSL amounts of pHRPTEpiCs (**A**) correspond to 5 × 10^6^ cells (orcinol stain), 2 × 10^6^ cells using the anti-Gb3Cer and anti-Gb4Cer antibody, respectively, and 6 × 10^5^ cells for the Stx1a and Stx2a TLC overlay assay, respectively. Applied GSL amounts of pHCoEpiCs (**B**) are equivalent to 5 × 10^6^ cells (orcinol stain), 5 × 10^5^ cells using an anti-Gb3Cer and anti-Gb4Cer antibody, respectively, and 1 × 10^6^ cells for the Stx1a and Stx2 TLC overlay assay, respectively. MHCs, monohexosylceramides. For further details refer to Detzner and collaborators [193,194], where the data were taken from.

In this section we present in condensed form the recently obtained novel data on the exact structures of Stx receptor GSLs detected in lipid preparations of primary human kidney and gut epithelial cells. For unambiguous identification of intrinsic cellular GSLs and to avoid any artefacts from the use of animal serum (known to contain GSLs, which can be incorporated from serum in the medium into the cellular plasma membrane), primary human renal proximal tubular epithelial cells (pHRPTEpiCs) were propagated for the purpose of GSL characterization with low serum and primary human colon epithelial cells (pHCoEpiCs) under serum-free conditions (pHRPTEpiCs could not be adapted to serum-free conditions). The synopsis of orcinol-stained neutral GSLs of pHRPTEpiCs and pHCoEpiCs together with the thin-layer chromatography (TLC) immunodetection of Gb3Cer and Gb4Cer with specific antibodies as well as with Stx1a and Stx2a directly on the surface of TLC plates with separated GSLs is shown in Figure 3. Detailed protocols of antibody- and Stx-mediated detection of TLC-separated globo-series GSLs using the TLC overlay technique have been recently published by us [195]. The presentation of the various lipoforms of Gb3Cer and Gb4Cer of pHRPTEpiCs and pHCoEpiCs completes this section.

As suggested from the orcinol-stained GSLs of pHRPTEpiCs (Figure 3A), kidney epithelial cells contain almost equal amounts of Gb3Cer, Gb4Cer, and Gb5Cer as prevalent GSLs. Anti-Gb3Cer and anti-Gb4Cer TLC overlay assays gave strongly stained double bands of Gb3Cer and Gb4Cer, harboring lipoforms with sphingosine (d18:1) and C24 fatty acyl chains in the respective upper bands and lipoforms with sphingosine (d18:1) and C16 fatty acyl chains in the respective lower bands, as the predominant GSL species [193]. Stx1a and Stx2a binding assays revealed Gb3Cer as the prevalent receptor for Stx1a and Stx2a, whereas Gb4Cer, the low affinity receptor of Stx1a and Stx2a, exhibited only weak binding intensity toward the Gb4Cer variant in the upper band where Gb4Cer with sphingosine (d18:1) and C24 fatty acid separates. Both Stx subtypes did not show any binding toward Gb5Cer.

The orcinol-stained GSLs in the chromatogram of pHCoEpiCs indicated a very low content Gb3Cer but a strongly stained double band of Gb4Cer (Figure 3B). This structure prediction corresponds to rather weakly anti-Gb3Cer and clearly anti-Gb4Cer positive immunostained bands. Stx1a and Stx2a exhibited a weak reaction with the small quantities of Gb3Cer as expected and recognized only the upper Gb3Cer lipoform with sphingosine (d18:1) and C24 fatty acid [194]. Similarly, only the upper band of the Gb4Cer doublet revealed a positive reaction with Stx1a and Stx2a, confirming the well-known low-binding potency of Gb4Cer toward these two Stx subtypes. Interestingly, pHCoEpiCs did not produce Gb5Cer, the Galβ3-elongated Gb4Cer structure found in pHRPTEpiCs (for GSL structures see Figure 2). This feature represents a striking difference between colon and kidney epithelial cells.

**Figure 4:**
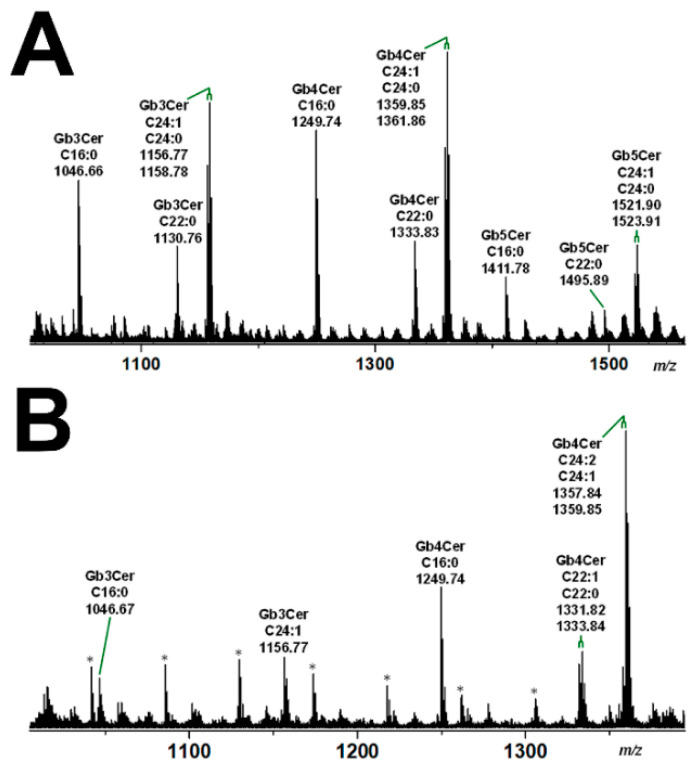
Overview MS^1^ spectra of the globo-series GSLs detected in the GSL preparations of pHRPTEpiCs (**A**) and pHCoEpiCs (**B**). The spectra show the various lipoforms of Gb3Cer, Gb4Cer, and Gb5Cer carrying sphingosine (d18:1) as the sole sphingoid base and variable fatty acyl chains as indicated. GSLs were detected as monosodiated species using the positive ion mode. The asterisks mark polyethylenglycols, which appear as serial contaminations in the GSL preparation of pHCoEpiCs. For further details refer to Detzner and collaborators [193,194], where the data were taken from.

The mass spectra obtained by electrospray ionization mass spectrometry (ESI MS), showing the various lipoforms of the detected GSLs of the globo-series of pHRPTEpiCs and pHCoEpiCs, are portrayed in Figure 4A,B, respectively. Prevalent Gb3Cer, Gb4Cer, and Gb5Cer lipoforms of kidney epithelial cells are those carrying sphingosine (d18:1) as the constant part of their ceramide cores linked with C16:0, C22:0, or C24:1/C24:0 fatty acyl chains (Figure 4A). Proofs of the MS^1^-characterized GSL structures were performed by collision-induced dissociation experiments (for details refer to Detzner and collaborators [193]). The MS^1^ spectrum obtained from a GSL preparation of colon epithelial cells is characterized by a lower extent of fatty acyl chain variability (Figure 4B) when compared to kidney epithelial cells. The dominant Gb3Cer and Gb4Cer lipoforms of colon epithelial cells harbor sphingosine (d18:1) as the sole sphingoid base of the ceramide lipid anchors linked to C16:0, C22:1/C22:0, or C24:1/C24:2 fatty acyl chains, whereas Gb3Cer and Gb4Cer lipoforms with C24:0 fatty acyl chains are missing (Figure 4B). Proofs of the MS^1^-characterized GSL structures were performed by collision-induced dissociation experiments (for details refer to Detzner and collaborators [194]). Collectively, pHRPTEpiCs exhibit a more complex profile with a higher extent of lipoform variability regarding the Stx receptors Gb3Cer and Gb4Cer and exhibit as a unique feature Gb5Cer, which is undetectable in pHCoEpiCs.

Interestingly, the Gb3Cer and Gb4Cer lipoforms of primary human kidney epithelial cells (pHRPTEpiCs) and primary human colon epithelial cells (pHCoEpiCs) as demonstrated in this review are very similar to those previously determined for primary human endothelial cells of brain and kidney as well as for erythroblast target cells having Cer (d18:1, C16:0), Cer (d18:1, 22:0), and Cer (d18:1, C24:1/C24:0) as their common lipid anchors [166,181,182,183,184]. Thus, functionally different cells, i.e., those lining the blood vessels and cells of the urinary tract system possess nearly the same collection of globo-series GSLs. Importantly, globo-series of the uroepithelial system can be abused by uropathogenic *E. coli*, which are known to adhere through their PapG adhesins to globo-series GSLs on the surface of human kidney and bladder uroepithelial cells [196].

## 5. Liquid-Ordered and Liquid-Disordered Membrane Phases

A fluid mosaic model for the gross organization and structure of proteins and lipids of biological membranes has been presented by Singer and Nicolson in 1972 [197]. However, the details of the lateral domain formation in biological membranes remained largely speculative at this time of membrane research. Since this fundamental work, enormous progress has been made considering the condensing effect of sterols on phospholipids spread as monomolecular films at the air–water interface and the thermodynamics of sphingomyelin-cholesterol interactions defining the biophysical model of the liquid-ordered and liquid-disordered membrane phase [198,199]. An updated model with emphasis on the mosaic nature of cellular membranes has been created regarding lipid–lipid, protein–protein, and lipid–protein interactions in the membrane plane as well as cell–matrix, cell–cell, and cytoskeletal interactions maintaining the unique mosaic organization of cell membranes in functional, dynamic domains [200,201,202]. Model membranes including unilamellar vesicles and lipid bilayers are emerging as essential tools for studying the lateral heterogeneity of the liquid-ordered and liquid-disordered membrane phase [203]. Importantly, fundamental similarities of liquid-ordered phase domains in unilamellar vesicles and detergent-resistant membranes, which are formed at lower temperatures by cooperative interactions of cholesterol with saturated acyl chains as well as unsaturated acyl chains in the presence of >25 mol% cholesterol, are known for a long time [204]. Thus, cholesterol exhibits multiple accommodating interactions with saturated acyl chains and less intensive interactions due to low miscibility with unsaturated acyl chains and transmembrane proteins [204]. The assembly of membrane domains with specific lipid and protein compositions regulates numerous biological processes underlying evolutionary design principles of phase segregation [205,206]. Sphingolipids may take center stage in re-defining their functional significance in the cholesterol-sphingomyelin-enriched liquid-ordered membrane phase, and a further type of microdomains termed ceramide-rich platforms with gel-like structure has been recently postulated offering some fresh view on the membrane architecture [205]. Sphingolipids are substantially involved in the formation of membrane microdomains in the glycerophospholipid bilayer that acts as a two-dimensional fluid construct allowing lateral movement of membrane constituents forming the lipid raft concept of membrane subcompartmentalization [207,208].

### 5.1. The Lipid Raft Concept

Lipid rafts are nano-scaled, heterogeneous, dynamic domains enriched in cholesterol, sphingomyelin, and GSLs creating a liquid-ordered phase with properties intermediate between a gel and fluid phase [199,209,210,211,212,213,214,215,216]. Such clusters of ordered lipids float freely within the liquid-disordered bilayer of cellular membranes profoundly influencing membrane organization and form dynamic platforms for the regulation of a plethora of vital cellular functions such as signal transduction, vesicular trafficking, protein processing, and membrane turnover in biological membranes [217,218,219,220].

GSL-cholesterol microdomains have been proposed in early reports to provide platforms for the attachment of lipid-modified proteins, such as glycosylphosphatidylinositol (GPI)-anchored proteins and src-family tyrosine kinases [221]. Cross-linking of GSLs as well as that of GPI-anchored proteins induce a rapid activation of src-family kinases and a transient increase in the tyrosine phosphorylation of several substrates, suggesting important roles of GSLs in signal transduction mediated by the microdomains [221]. Such signaling platforms in the plasma membrane are instrumental for the translation of the extracellular cues into intracellular signals for gene activation. To this end, different membrane-bound components need to be assembled in a coordinated manner to exert cellular communication as it is the case in diverse raft-based signaling pathways of T lymphocytes and natural killer cells [222]. The structure of lipid rafts is dynamic, resulting in an ever-changing assembly of membrane constituents with cholesterol as the major compound for formation and configuration of lipid raft microdomains, which provide signaling platforms capable of activating both pro-apoptotic and anti-apoptotic signaling pathways [223]. Raft microdomains exist as caveolae, morphologically recognizable flask-like invaginations, or as less easily detectable planar forms furthermore characterized by their interaction with and dynamic rearrangement of cytoskeletal components [224]. The interaction between microdomains and the underlying cytoskeleton regulates many facets of eukaryotic cellular functions and cellular adaptation to changing environments. Moreover, many processes necessary for the correct functions of the nervous system occur in lipid rafts and are dependent on lipid raft organization [225,226]. Lipid rafts are deeply involved in molecular transcytosis, a well-known process concerning the transport of metabolites between the apical and basolateral faces of various cell types, and maintenance of cellular homeostasis [227]. An increasing body of evidence indicates a substantial role of lipid rafts in the modulation of immune signaling and its potential to combat autoimmune diseases and inflammatory disorders [228]. The involvement of lipid rafts and caveolae in endothelial cell membrane biogenesis and cell response to extracellular stimuli, endothelial cell migration and proliferation as well as angiogenesis and maturation of the blood vessels are key issues in the organization of the human endothelium lining the different vascular beds [229,230]. The apical cell membrane of absorptive intestinal enterocytes and kidney proximal tubule epithelial cells is formed as a brush border, composed of regular, dense arrays of microvilli. The microvillar surface is organized in cholesterol/sphingolipid-enriched atypical lipid rafts with specialized functions, which exhibit, in particular those of enterocytes, some unusual properties [231]. These include stable rather than transient/dynamic microdomains resisting solubilization with Triton X-100 at physiological temperature and pleiomorphic, deep apical membrane invaginations between adjacent microvilli. The architecture of these rafts supports a digestive and/or absorptive strategy for nutrient assimilation suggesting lipid rafts as pluripotent microdomains capable of adapting in size, shape, and content to specific cellular functions [232].

### 5.2. Lipid Raft Association of Stx-Binding GSLs

It is widely acknowledged that attachment of Stxs to GSLs in the outer leaflet of the plasma membrane, followed by internalization and retrograde routing of the toxin-GSL-complex to intracellular targets, is favored by lipid raft-associated GSLs [3,233]. The arrangement of GSL receptors in lipid rafts has been reported to be a pivotal requirement for efficient binding and internalization of Stxs [234,235]. Through multivalent binding to GSLs, Stxs induce lipid clustering and negative membrane curvature, which drives the formation of inward membrane tubules [236,237,238]. The presence of the Stx receptor GSL Gb3Cer in lipid rafts is believed to play a key role in the pathology of HUS since binding of Stx1a and Stx2a to tissue sections of human renal glomeruli was found detergent-resistant, whereas Stx binding to renal tubules was detergent sensitive [239,240]. Furthermore, fatty acid heterogeneity of different Gb3Cer lipoforms may have a functional role regarding the membrane-organizing principle of lipid rafts and perhaps in the pathogenic outcome of HUS [234]. Anyway, presence of Gb3Cer within membrane microdomains in glomerular cells may be the basis for the glomerular-restricted pathology of Stx-induced HUS [234,240]. Another approach indicated the requirement of lipid rafts for the uptake of Stx1(a) across the apical membrane of Caco-2 cells as previously shown [241], whereby not only presence of Gb3Cer but also the density of Gb3Cer in lipid rafts may be important for Stx binding as shown for Vero cells [242].

### 5.3. Detergent-Resistant Membranes as Membrane Analog Tools

Increased content of cholesterol and sphingolipids in lipid rafts renders such clusters relatively robust against solubilization by non-ionic detergents, allowing isolation of detergent-resistant membranes (DRMs) and accompanying nonDRMs by sucrose density gradient ultracentrifugation [243,244]. DRMs and nonDRMs are used in many fields as equivalents for the liquid-ordered and liquid-disordered membrane phase, respectively [213]. DRMs can readily be prepared and exhibit many properties such as lipid rafts [245,246,247,248]. DRMs have been successfully applied in the analysis of Stx-receptor interactions and retrograde trafficking of the toxin and the association of Stx with DRMs has been reported as an essential requirement for a cytotoxic effect [242,249].

### 5.4. Membrane Distribution of Stx Receptor GSLs in pHRPTEpiCs and pHCoEpiCs

**Figure 5:**
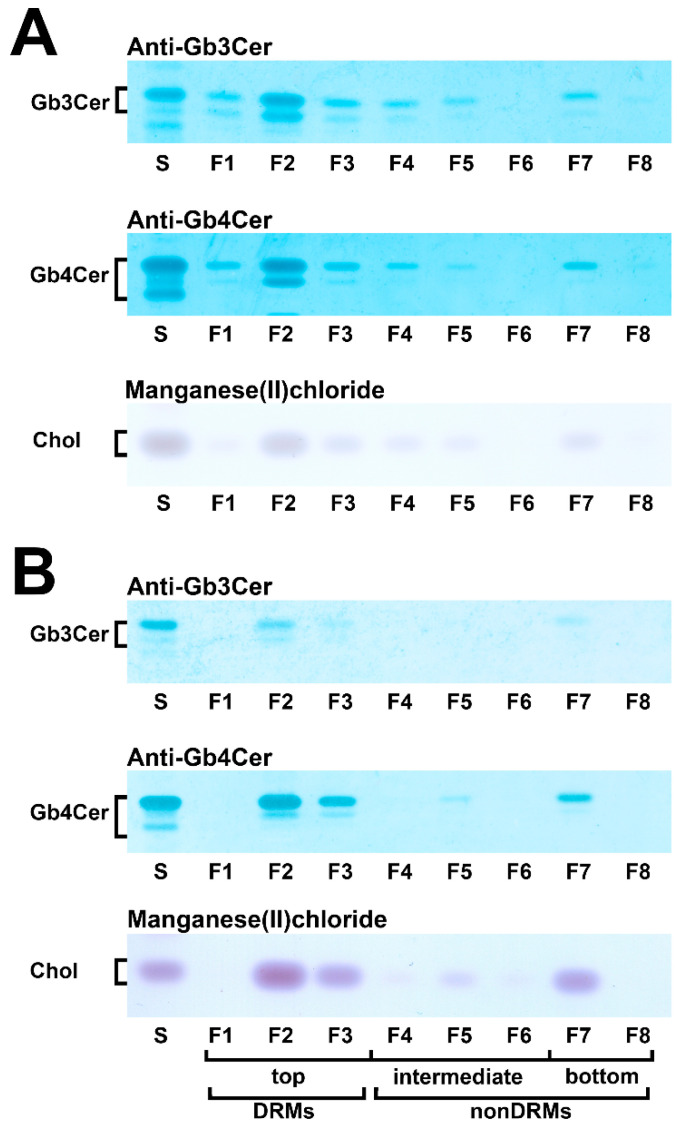
Distribution of Gb3Cer, Gb4Cer, and cholesterol to sucrose gradient fractions F1 to F8 prepared from pHRPTEpiCs (**A**) and pHCoEpiCs (**B**). Anti-Gb3Cer and anti-Gb4Cer antibodies were employed for immunostaining and manganese(II)chloride for cholesterol (Chol) detection. Standard (S) equivalents of 2 µg and 0.2 µg of reference neutral GSLs from human erythrocytes and 1 µg of reference cholesterol (Chol) were applied as positive controls for the anti-Gb3Cer and anti-Gb4Cer TLC overlay assay and the cholesterol detection, respectively. DRMs, detergent-resistant membranes. For further details refer to Detzner and collaborators [193,194], where the data were taken from.

With the aim to gain basic knowledge about the membrane distribution of the Stx receptor GSLs of primary human colon and renal epithelial cells, it makes sense to investigate the distribution of Gb3Cer and Gb4Cer, the high and low affinity Stx receptor, respectively, in connection with the “membrane glue” cholesterol of primary human colon and renal epithelial cells using lipid raft-analog membrane preparations. To this end the distribution of Gb3Cer and Gb4Cer to DRM (top) and nonDRM (below) fractions obtained from sucrose gradient preparations of kidney proximal tubular and colon epithelial cells was analyzed in TLC overlay assays with anti-Gb3Cer and anti-Gb4Cer antibodies, respectively, and the detection of cholesterol was performed with manganese(II)chloride as shown in Figure 5. DRMs (F1 to F3) represent the top fractions of the sucrose gradients and those below are the nonDRMs (F4 to F7), which can be further subdivided into intermediate (F4 to F6) and bottom fractions (F7 and F8). Gb3Cer and Gb4Cer added up to 78% and 81%, respectively, in the three DRM fractions (F1 to F3) and to a corresponding lower relative content of 23% and 19% in the five nonDRM fractions (F4 to F8) of pHRPTEpiCs along with 67% and 33% of cholesterol in DRMs and nonDRMs, respectively (Figure 5A) [193]. Similar distribution patterns were obtained in case of pHCoEpiCs with Gb3Cer and Gb4Cer summed up to 80% and 79%, respectively, in the DRM fractions (F1 to F3) and to a related lower relative content of 20% and 21% in the nonDRM fractions (F4 to F8) of pHCoEpiCs together with 69% and 31% of cholesterol in DRMs and nonDRMs, respectively (Figure 5B) [194].

**Figure 6:**
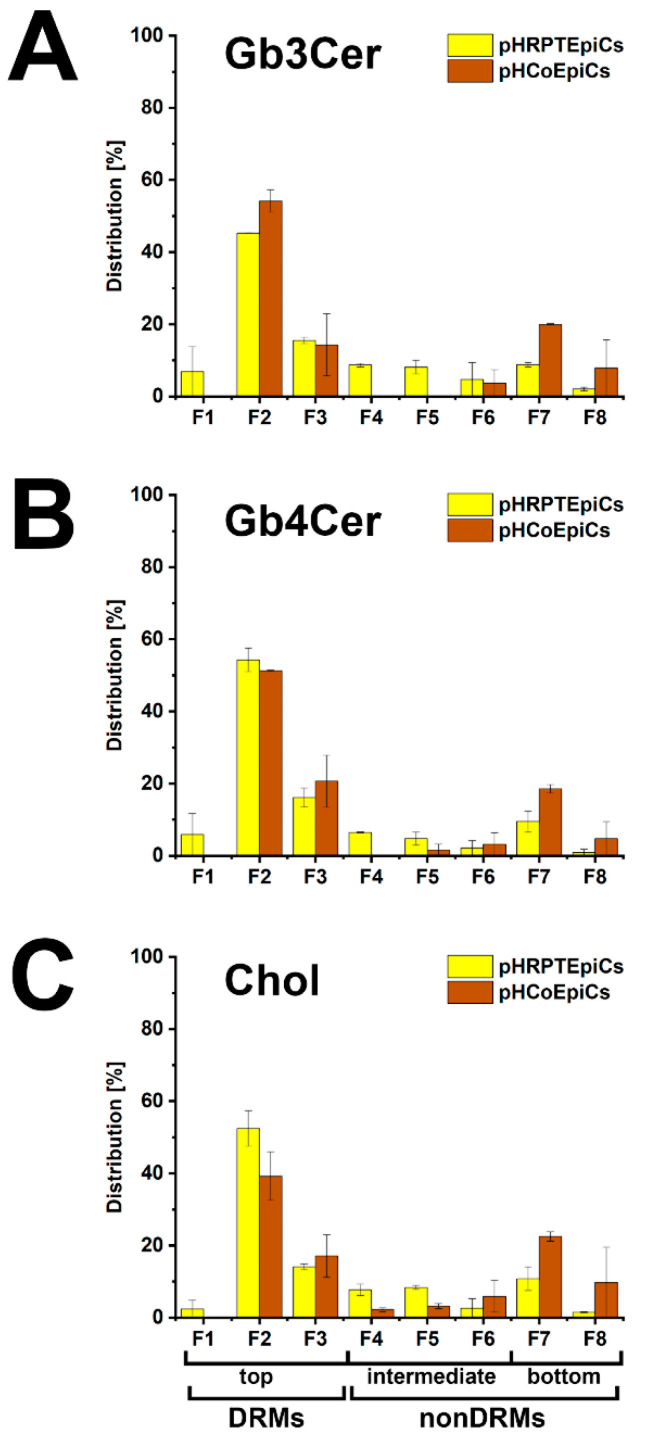
Bar chart illustration of the distribution of Gb3Cer (**A**), Gb4Cer (**B**), and cholesterol (**C**) to sucrose gradient fractions F1 to F8 obtained from pHRPTEpiCs and pHCoEpiCs. The immunostained TLC bands of Gb3Cer and Gb4Cer and the cholesterol spots shown in Figure 5 were densitometrically quantified and normalized for each fractionation to 100%. For further details refer to Detzner and collaborators [193,194], where the data were taken from.

The bar chart depicted in Figure 6 illustrates the distribution of Gb3Cer, Gb4Cer, and cholesterol to the DRMs and nonDRMs showing a high degree of resemblance of distribution patterns for the analytes of pHRPTEpiCs (yellow bars) and pHCoEpiCs (brown bars). The preference of Gb3Cer and Gb4Cer to the DRM fractions, particularly to the canonical DRM fraction F2, suggests enrichment of the Stx receptor GSLs in the liquid-ordered membrane phase of lipid rafts. Moreover, Gb3Cer and Gb4Cer together with cholesterol can be taken as true microdomain markers not only of the DRM fractions but also for the liquid-ordered membrane phase of lipid rafts.

## 6. Different Susceptibility of Human Kidney and Colon Epithelial Cells toward Stx1a and Stx2a

The general susceptibility of primary human renal epithelial cells toward Stx has been documented in previous reports [250,251,252,253,254,255,256]. Evidence has been provided that kidney epithelium harbors, besides the renal endothelial microvasculature, further targets of Stx suggesting epithelial contribution to clinical signs of HUS [159,191,257,258,259] and involvement in Stx-mediated kidney failure as previously shown in a mouse model [260,261].

The situation for colon epithelial cells with regard to Stx-mediated cell-damaging effects is less clear compared to renal epithelial cells. The basic susceptibility of primary intestinal epithelial cells toward Stx is considered certain as reported in a number of publications [89]. However, tumor-derived colonic epithelial cell lines such as Caco-2, HCT-8, HT-29 and T84 were predominantly used in most previous studies as epithelium equivalent, although their susceptibility toward Stx and cellular endowment with Stx receptors might strongly differ from that of primary colonic epithelial cells [126,262,263,264,265,266,267,268,269]. Of note, despite partly contradictory results in the case of the T84 cell line [126], these studies were helpful, because they indicated the principal capability of colon cancer cell lines to produce Stx-binding GSLs of the globo-series and showed susceptibility toward Stx suggesting colon epithelial cells as possible targets for Stx that may be directly damaged by the toxin in the intestine. At that time the common assumption was that normal human colonic epithelial cells lack the Stx receptor GSL Gb3Cer [126,266,270], although contrary data suggested that Gb3Cer may be present in small quantities in human colonic epithelia, where it may compete for Stx binding with the more abundant GSL Gb4Cer [268]. In addition, Stx-binding and presence of Gb3Cer have been detected histologically in normal intestine [270]. Thus, the existence of the Stx receptor GSLs in primary human colon epithelial cells has been and may be still a matter of debate [89].

Primary cells in general preserve much better the genetic signature of normal cells of healthy donors than tumor-descendants isolated from cancer patients. Like other primary cell types, primary human renal proximal tubular epithelial cells (pHRPTEpiCs) and primary human colon epithelial cells (pHCoEpiCs) have a limited life span when propagated in vitro and require certain growth factors and/or other supplements when compared to unlimited growing immortal cell lines [271,272]. From our side, the number of passages of primary kidney and intestinal epithelial cells is recommended to be less than ten passages for physiological experiments. Unambiguous signs of senescence are the formation of a spindle-like phenotype and/or the loss of the characteristic cobblestone-like morphology accompanied with a reduced proliferation rate that indicate cellular dedifferentiation. Immediately after receipt a master bank of the primary cells should be set up. Cells at very early passages should be stored as deep frozen aliquots in the gas phase over liquid nitrogen at approximately −192 °C. Cells should be thawed on demand, cultivated only for the shortest possible period of time and discarded when reaching passage 10 of cultivation. We used primary human kidney epithelial cells and primary human colon epithelial cells and summarize comparatively in this section the recently acquired data about their susceptibility toward the clinically EHEC-HUS-relevant Stx-subtypes Stx1a and Stx2a [193,194].

**Figure 7:**
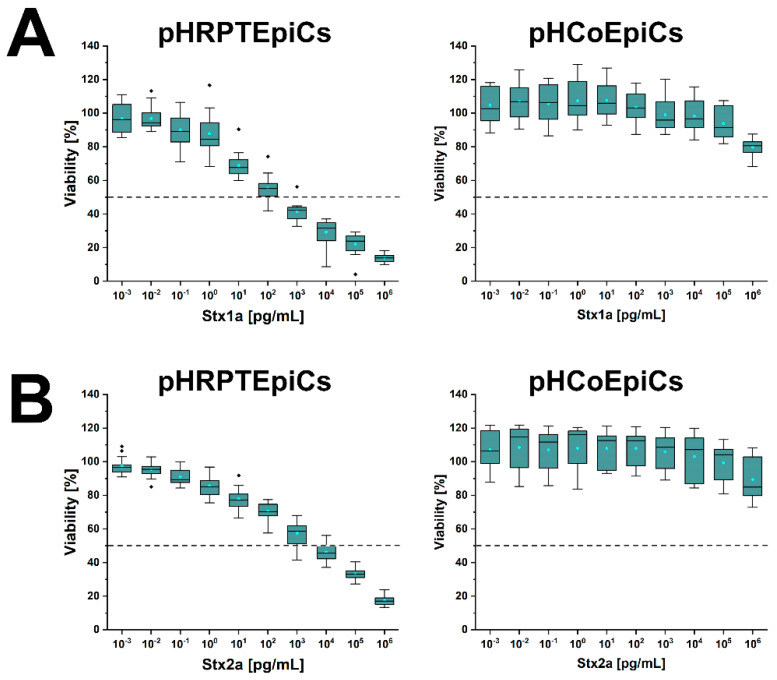
Cytotoxic response of pHRPTEpiCs and pHCoEpiCs upon exposure to increasing concentrations of Stx1a (**A**) and Stx2a (**B**). The cell-damaging effect was determined with the crystal violet assay, and the absorption readouts of Stx1a- and Stx2a-treated cells are displayed as box plots. The extent of cell damage is given as percentage values related to untreated cells, which represent 100% viable cells. For further details refer to Detzner and collaborators [193,194], where the data were taken from.

Figure 7 shows the course of the survival rates of pHRPTEpiCs from the kidney upon exposure to increasing concentrations of Stx1a (Figure 7A, left panel) and Stx2a (Figure 7B, left panel) in comparison to Stx1a- and Stx2a-treated pHCoEpiCs of the colon (Figure 7A,B, right panel, respectively). A significant initial sensitivity of pHRPTEpiCs toward Stx1a occurred at a toxin concentration of 10^0^ pg/mL exerting a decrease in the cell survival rate to 92.1 ± 10.6% as shown in the box plot (Figure 7A). The concentration-dependent gradually reduced viability of the cells dropped to 12.8 ± 1.9% viability when challenged with the highest applied Stx1a concentration of 10^6^ pg/mL (equivalent to 1 µg/mL). The 50% cytotoxic dose (CD_50_) of Stx1a amounted to 1.31 × 10^2^ pg/mL for pHRPTEpiCs. On the other hand, the course of the cell survival rate showed high tolerance of colon epithelial cells toward Stx1a with only marginal response to toxin concentrations in the range of 10^3^ to 10^5^ pg/mL (Figure 7A, right panel). Application of the highest toxin concentration of 1 µg/mL of Stx1a resulted in a reduced cell viability of 79.7 ± 5.1%.

A stepwise rise in the cytotoxic action was detected after exposing pHRPTEpiCs to Stx2a (Figure 7B, left panel) indicating a beginning toxin-mediated decrease in viability at an Stx2a concentration of 10^−1^ pg/mL (90.9 ± 5.6% viability) and a continuing decline to a final cell survival of 18.3 ± 2.8% when treated with 10^6^ pg/mL (equivalent to 1 µg/mL) of Stx2a. The CD_50_ of Stx2a for pHRPTEpiCs accounted for 1.66 × 10^3^ pg/mL. When probing pHCoEpiCs with Stx2a, a slight susceptibility was recognized for Stx2a corresponding to a cell survival of 89.5 ± 12.4% upon toxin challenge using the uppermost toxin concentration of 1 µg/mL applied in this study (Figure 7B, right panel).

In sum, Stx1a exhibited a more than one order of magnitude higher cytotoxic activity against renal pHRPTEpiCs than Stx2a (CD_50_ Stx1a of 1.31 × 10^2^ versus CD_50_ Stx2a of 1.66 × 10^3^ pg/mL) based on the comparison of the 50% cytotoxic doses indicating a more efficient cell killing rate of Stx1a. In striking contrast, intestinal pHCoEpiCs were largely refractory in a concentration range of 10^−3^ up to 10^5^ pg/mL of both Stx subtypes. However, colonic epithelial cells were not resistant to the toxins showing at least a slight cell damage upon application of the uppermost concentration of 1 µg/mL of both Stx subtypes applied in these assays [193,194]. The extremely low susceptibility of primary colonic epithelial cells suggests a large resilience of the intestinal epithelium against the human-pathogenic Stx1a- and Stx2a-subtypes due to the low content of the high-affinity Stx-receptor Gb3Cer in colon epithelial cells when compared to the high susceptibility and higher content of Gb3Cer of primary renal epithelial cells.

## 7. Therapeutic Options of EHEC Infections

Despite decades of research elucidating the mechanisms of Stx-mediated toxicity, there is no specific and effective remedy to date for curation of patients suffering from EHEC-associated HUS. Therapy mostly relies on supportive intensive care regimens for both EHEC-associated intestinal and potentially lethal extraintestinal complications [152,160,273,274,275]. Importantly, differential features arise in an inconsistent patient population between pediatric and adult clinical presentation. EHEC-HUS in adults is marked by prevalence of neurological symptoms and a poorer prognosis when compared to children having consequences for critical care management and treatment of the disease that remains a public threat due to lack of a specific treatment [276]. Although early diagnosis and supportive therapy are essential to limit complications and supportive care is beneficial for patients and has significantly reduced the mortality rate [152,160,277], the emergence of new strains with increased aggressive virulence potential requires clinical and research initiatives of high public health priority using novel molecular typing systems such as whole genome sequence-based approaches for unravelling emerging new pathotypes in more detail [59,278,279,280,281,282,283,284].

### 7.1. Application of Antibiotics or Not That’s the Question

The administration of antibiotics in EHEC infections was and remains controversial because of concerns about triggering HUS by increasing Stx production [285,286,287]. Stxs are encoded by genes located on genomes of lambdoid prophages and certain antibiotics stimulate their induction leading to enhanced production of Stxs [288]. Although numerous studies have reported that antibiotics enhance the severity of disease symptoms and increase the risk of progression to HUS development, further corroborated by in vitro antibiotic studies using certain EHEC strains, others have reported that antibiotics do not have any effect or can even reduce the rate of HUS development in EHEC infections [289,290,291,292,293,294,295,296]. The current data situation leads to the conclusion that the infecting EHEC strain, the type of antibiotic, and the timing of its application appear to significantly affect the development of HUS in EHEC-infected patients [285].

### 7.2. Development of Non-Antibiotic Therapeutics

In recent years, a variety of alternative treatment approaches and therapeutic interventions has been developed and evaluated in vitro, in animal models and clinical trials for preventing EHEC-associated HUS [297,298]. The majority of possible non-antibiotic therapeutics has been or is in the developmental stage aimed to neutralize Stx, to prevent toxin adhesion, to block receptor biosynthesis, and to interfere trafficking, processing, and activity of the toxin within the cell [6,156,293,299,300,301]. Since Stx induces the secretion of inflammatory cytokines and chemokines from susceptible cells that contribute to the pathogenesis of HUS, these compounds are useful indicators of disease activity as well as predictors of disease progression and candidates for an anti-inflammation therapy as an additional treatment regimen for severe *E. coli*-associated HUS [302].

#### 7.2.1. Inhibitors of Glycosphingolipid Biosynthesis and Stx-Neutralizing Glycoconjugates

Ceramide is the hydrophobic backbone of all complex amphipathic glycosphingolipids (GSLs). Its initial glycosylation forming glucosylceramide (GlcCer) is the first committed and rate-limiting step in the biosynthesis of GSLs with GlcCer core leading to the various GSL-families including the globo-series [303]. A number of ceramide analogs such as classical D-PDMP and many others has been scrutinized in the past as potential inhibitors of GlcCer synthase mainly developed for the treatment of human lipid storage diseases named as substrate reduction therapy [304,305,306,307,308]. The capability of traditional and novel GlcCer synthase inhibitors to reduce the cellular level of the Stx receptor Gb3Cer in various cell types including human epithelial and endothelial cells [183,309] and to prevent the cytotoxic action toward this way Gb3Cer-truncated target cells has expedited an additional focus on the Stx receptor Gb3Cer as therapeutic target in Stx-mediated HUS. An example of a newly developed GlcCer-synthase inhibitor is the ceramide analog Eliglustat [308], also primarily developed as an alternative approach to the enzyme replacement therapy of patients suffering from GSL storage diseases [310], effectively protects human renal tubular epithelial cells from Stx-caused cellular damage due to reducing the cellular Gb3Cer levels suggesting its potential as Stx protector [311,312]. The prevention of Gb3Cer-synthesis and neutralization of Stx-mediated cytotoxic action by the ceramide analog C-9, shown for primary human renal epithelial cells in vitro and an in vivo animal HUS model in rats offer a further option for treatment of EHEC-HUS [255,313].

Aligned to the opposite site of an amphipathic GSL from the lipid anchor, modifications of the hydrophilic glycan represent a further approach to impede or prevent Stx binding. Such metabolic modification can easily be done and has been reported for feeding of in vitro propagated cells with 2-deoxy-D-glucose or 2-fluoro-2-deoxy-D-glucose revealing protective effects of both compounds against Stx [314,315]. 2-deoxy-D-glucose becomes incorporated into the carbohydrate moiety of GSLs and protects cells against Stxs [314], while 2-fluoro-2-deoxy-D-glucose inhibits GlcCer biosynthesis thereby reducing the cellular levels of GSLs as shown for various cell types including human brain microvascular endothelial cells. This glucose-modification is much more efficient in protecting cells against Stx when compared to 2-deoxy-D-glucose [315]. Furthermore, the clinically approved glucose-derivative Miglustat has been shown being effective in human endothelial and epithelial cells to decrease the level of Stx receptor Gb3Cer suggesting its application as a feasible strategy to protect kidney tissues from Stx-mediated kidney injury [316]. Collectively, the enumerated ceramide analogs and glucose-derivatives suggest potential clinical applications for Stx-caused diseases.

Since protein toxins of enterotoxic bacteria have proven to be attractive targets for drug development [300,317], numerous therapeutic glycoconjugates based on Stx-specific analogs of the glycan receptor Gb3 have been developed [297,318]. Synsorb Pk [319], Starfish [320], Daisy [321], SUPER TWIGS [322,323], polymeric acrylamide-Gb3 conjugates [324], Gb3 (glycan) encapsulated gold nanoparticles [325,326], neoglycolipid-spiked glycovesicles [327,328] or engineered probiotics expressing Gb3 analogs on their surface [329] are examples of glycoconstructs, developed for neutralization of Stxs as described more precisely in a nice and highly recommended recent review [297]. However, although effective in vitro, potential Stx-binding neutralizers have failed in vivo showing no benefit in clinical trials and none of them has received clinical approval to date [191,292].

#### 7.2.2. Monoclonal Antibodies

Despite a tremendous increase of knowledge has been gained with regard to the generation of neutralizing humanized (chimeric) or human monoclonal anti-Stx antibodies to combat Stx-mediated diseases [330,331,332], so far no monoclonal antibody against Stx1(a) or Stx2(a) has received clinical approval [297,333]. The broadly administered anti-C5 monoclonal antibody Eculizumab during the 2011 outbreak of an O104:H4 EHEC strain in Germany gave an equally good outcome of treated versus untreated patients and pointed to an advantageous use, at least for severe cases [334]. This anti-C5 complement blocker has obviously made the difference between favorable or detrimental outcome [334,335]. The administration of Eculizumab in EHEC-associated HUS with neurological involvement indicated that early use of Eculizumab appeared to improve neurological outcome, whereas late treatment seemed to show less benefit suggesting advantage of prophylactic Eculizumab therapy before development of neurological symptoms [336]. Thus, treatment of EHEC-HUS patients with Eculizumab has shown positive clinical improvement and proven effective in some cases [191,337,338].

#### 7.2.3. Further Alternative Therapeutic Concepts

Among further alternative therapeutic strategies, a promising approach is the use of probiotic microorganisms showing antagonistic effects on EHEC strains of various serotypes [292,339,340,341]. Suitable vaccine candidates against EHEC infections are polysaccharide conjugates such as constructs built up from *E. coli* O157 or *E. coli* O145 polysaccharides linked to bacterial carrier proteins offering high prospects for effective preventive treatment for future clinical studies [342,343]. Phage therapy using specific phages against *E. coli* O157:H7 has to be taken into consideration as well. More than 60 specific phages are known so far and in vitro experiments have been successful in elimination or reduction of *E. coli* O157:H7 numbers, but in vivo experiments have not been as promising [344]. The proof or principle of the novel antibiotic-peptide wrwycr has been reported effective in killing of EHEC in synergistic combination with antibiotic treatment without enhancing release of Stxs. This strategy offers a potential new candidate for a preventive antimicrobial for EHEC infections [345,346]. The retrograde transport of internalized Stx directly from early endosomes to the Golgi apparatus is an essential step to bypass degradation in the late endosomes and lysosomes, which then continues to the endoplasmic reticulum before translocation of the enzymatically active moiety to the ribosomal target in the cytosol [167,301,309,347,348,349,350]. This renders the crucial retrograde transportation route an ideal attack point for small molecule inhibitors of toxin trafficking as possible therapeutics acting at the endosome/Golgi interface [351,352]. Substances that interfere with intracellular trafficking inhibiting the transport of Stx have been summed in recent reviews [6,297] and will not be discussed further at this point.

#### 7.2.4. Current Situation

A comprehensive study that summarized the results of clinical trials for preventing EHEC-associated HUS including antibiotics, the Stx inhibitor Synsorb Pk, and a monoclonal antibody against Stx (Urtoxazumab) revealed no firm conclusions about the efficacy of these interventions given the small number of included studies and their small sample sizes [298]. Collectively, despite significant advances in understanding the molecular mechanisms of Stx being imperative for the design of appropriate drugs or adjunctive therapeutics, a rationally designed drug that targets Stx has yet not reached the market [191,297,298].

## 8. Outlook

Understanding of the Stx-mediated cellular injury requires basic knowledge on the molecular mechanisms triggering the interaction between the toxin and its receptor molecules. The GSL receptors of Stx exhibit a huge variety regarding their lipid membrane anchor videlicet the ceramide moiety. The functional impact of the various lipoforms carrying C16 up to C24 fatty acyl chains and their enrichment and/or specific localization in a certain lipid environment in the liquid-ordered membrane phase known as lipid rafts are still enigmatic. This applies equally to endothelial cells [166] and epithelial cells as summarized in this review exhibiting a high variability of Gb3Cer and Gb4Cer species with Cer (d18:1, C16:0), Cer (d18:1, C22:0/C22:1), and Cer (d18:1, C24:0/C24:1). The biological function of this ceramide heterogeneity is so far largely unclear and remains to be resolved in the future. However, from our data it is tempting to speculate that the long-chain C24 fatty acyl chains of Gb3Cer and Gb4Cer found in human endothelial cells [166], human erythroblasts [181,184], and human kidney and colon epithelial cells [192,193,194] may be involved in a molecular interdigitation of outer membrane acyl chains with inner membrane phospholipids, a process known as “handshaking”. However, this molecular interaction has been so far described for sphingomyelin with C24 fatty acyl chain located in the outer layer of the plasma membrane with the glycerophospholipid phosphatidylserine (18:0/18:1) in the inner layer of the plasma membrane [187,188]. Collectively, unravelling the functional role of the diversity of GSL lipoforms in toxin-membrane interactions and their molecular arrangement in native lipid rafts of the plasma membrane still remain challenging tasks for glycobiologists and requires further in-depth investigations in the future to solve these puzzles.

Groundbreaking advancements achieved with newly developed matrix-assisted laser desorption/ionization mass spectrometry (MALDI MS) imaging technologies allows for label-free in situ detection of glycolipids and further lipids in tissue or organ sections [353,354,355,356]. MS imaging can simultaneously record the lateral distribution of numerous biomolecules in tissue slices [357] and provides precise structural details of membrane constituents on the cellular niveau with subcellular high resolution in mass and space at the single-cell level [358,359,360,361]. On-tissue enzyme treatment can overcome ion suppression effects of bulk phospholipids for enhanced MS imaging of GSLs [362] and chemical modification has been demonstrated to allow the localization of carbon-carbon double bonds in phospholipids and glycolipids [363,364]. Moreover, imaging mass spectrometry has the power of a diagnostic tool for resolving lipid-related pathological conditions, which can be combined with conventional (immuno) histochemical staining and immunofluorescence microscopy to increase our understanding of a pathological development versus the healthy state [365,366,367]. Thus, the analysis of glycolipids in tissue sections of kidney, brain, or cancer tissue can shift our understanding of human health to a higher level [367,368]. Further aspects of future MS research are geared to the development of three-dimensional MS imaging aimed at studying the topographic distribution of compounds on irregular 3D surfaces with subcellular resolution and to unravel lipid heterogeneity of cell states during differentiation by coupling high-resolution mass spectrometry imaging to single-cell lipidomics to gain new insights into the self-organization of multicellular systems [369,370,371,372].

Progress in understanding the protein-carbohydrate dependent pathogen–host interaction is the basis for the manufacture of novel glycotherapeutics basically postulated by the pioneers of glycoscience more than two decades ago [373,374]. Carbohydrate-based multivalent inhibitors have already been created in the early phase of glycoresearch and in the following years with a few showing the potential to competitively inhibit the binding of Stx to glycosides with Gb3-core and to neutralize its cytotoxic activity in vitro [320,327,328,375,376,377,378,379]. Real-time interaction analysis of GSLs in model membranes and membrane preparations with lectins of all kinds, including Stxs and viral hemagglutinins, employing the recently applied surface acoustic wave (SAW) technology [170,380,381] combined with inhibition assays using, e.g., multivalent glycan derivatives, has a promising perspective for the development of anti-adhesion therapeutics. On the other hand, GSL reduction therapy based on GSL-lowering agents previously developed for the treatment of GSL storage disorders (glycosphingolipidoses) [306], can be used to prevent and/or combat infections [382]. Noteworthy in this context, selective elimination of uropathogenic *E. coli*, endowed with type 1 fimbriae carrying the mannose-binding FimH adhesin, from the intestine using a high-affinity inhibitory mannoside (M4284), has been shown as a functional blocker of the pathogen [383], already predicted by Nathan Sharon more than 15 years ago [376]. However, current approaches to the treatment of diseases that have their origin in the attachment of pathogens and/or their virulence factors to certain glycan structures exposed on the surface of target cells are still in their infancy.

Alternatives to competitive Stx blockers are small molecules that act directly through anti-toxin properties or indirectly on the toxin by preventing toxin expression and are in the sights of new research approaches [6,384]. Moreover, certain cell permeable agents are capable to intracellularly interfere with the retrograde cargo of Stx and can efficiently target the toxin’s early endosome-to-Golgi transport acting as inhibitors of the trafficking step that makes them potential therapeutics [351].

The role of epithelial cells of colon and kidneys in the infection process of EHEC and particularly their involvement in the development of HUS are currently discussed intensively. We have therefore given priority in this review to summarizing our latest findings on the existence and relative abundance as well as the exact structures of the distinct Stx receptor GSLs in primary human kidney and colon epithelial cells. Both epithelial cell types possess a set of various lipoforms of Gb3Cer (high-affinity Stx receptor) and Gb4Cer (low-affinity Stx receotor) each harboring ceramides with sphingosine (d18:1) as the sole sphingoid base and variable fatty acyl residues with C16, C22 and C24 chain length. However, the main differences were the low level of Gb3Cer in primary colonic epithelial cells when compared with primary renal epithelial cells and the presence of Gb5Cer in kidney epithelial cells, while colon epithelial cells are lacking this GSL. While the role of Gb5Cer is irrelevant for Stx-GSL interaction—Stx1a and Stx2a do not bind to Gb5Cer—The low content of Gb3Cer correlated with low sensitivity of colonic epithelial cells toward both HUS-associated Stx subtypes. This fact may explain the resilience in situ of the colon epithelium toward EHEC-released Stx in the intestine, whereas strong Stx-mediated cellular damage of the renal epithelium correlates with the high content of globo-series GSLs particularly of Gb3Cer and thus with the clinical signs of kidney damage in the manifestation of HUS. Gb3Cer and Gb4Cer mainly distributed among detergent-resistent membranes used as lipid raft-analog supramolecular structures, suggesting a functional role of both Stx receptor GSLs in the liquid-ordered membrane phase of the plasma membrane of epithelial cells in the kidney and the colon. These findings may help to clarify some critical points that were (and might still be) a matter of debate regarding the involvement of renal and colonic epithelial cells in the development of HUS. In conclusion, it should be noted that the “traditional” supportive therapy of HUS can hopefully be replaced in the near future by alternative treatment approaches including the application of anti-Stx monoclonal antibodies, toxin receptor analogs, or vaccination strategies, which have been evaluated so far using in vitro and animal models, of which a few have progressed to the clinical trial phase [297].

## Data Availability

Not applicable.
